# Are anti-SARS-CoV-2 S/N IgG/IgM antibodies always predictive of previous SARS-CoV-2 infection?

**DOI:** 10.1515/almed-2023-0008

**Published:** 2023-03-24

**Authors:** Giuseppe Lippi, Brandon M. Henry, Laura Pighi, Simone De Nitto, Gian Luca Salvagno

**Affiliations:** Section of Clinical Biochemistry, University of Verona, Verona, Italy; Clinical Laboratory, Division of Nephrology and Hypertension, Cincinnati Children’s Hospital Medical Center, Cincinnati, OH, USA; Service of Laboratory Medicine, Pederzoli Hospital, Peschiera del Garda, Italy

**Keywords:** antibodies, COVID-19, infection, SARS-CoV-2, serology

## Abstract

**Objectives:**

We planned this study to verify whether immunoassays for quantifying anti-SARS-CoV-2 IgG/IgM antibodies against both spike (S) and nucleocapsid (N) proteins may be used for identifying previous SARS-CoV-2 infections.

**Methods:**

The study population consisted of a cohort of fully vaccinated healthcare workers. All study subjects underwent regular medical visits and molecular testing for diagnosing SARS-CoV-2 infections every 2–4 weeks between 2020–2022. Venous blood was drawn for measuring anti-SARS-CoV-2 antibodies with MAGLUMI 2019-nCoV lgG/IgM CLIA Assays directed against both SARS-CoV-2 S and N proteins.

**Results:**

Overall, 31/53 (58.5%) subjects had tested positive for SARS-CoV-2 by RT-PCR throughout the study (24 once, 7 twice). No positive correlation was found between anti-SARS-CoV-2 S/N IgM antibodies and molecular test positivity. In univariate regression analysis, both a molecular test positivity (r=0.33; p=0.015) and the number of positive molecular tests (r=0.43; p=0.001), but not vaccine doses (r=−0.12; p=0.392), were significantly correlated with anti-SARS-CoV-2 S/N IgG antibodies. These two associations remained significant in multiple linear regression analysis (p=0.029 and p<0.001, respectively) after adjusting for sex, age, body mass index, and vaccine doses. In ROC curve analysis, anti-SARS-CoV-2 S/N IgG antibodies significantly predicted molecular test positivity (AUC, 0.69; 95% CI; 0.55–0.84), with the best cutoff of 0.05 AU/mL displaying 67.9% accuracy, 0.97 sensitivity, and 0.27 specificity.

**Conclusions:**

Although anti-SARS-CoV-2 S/N IgG antibodies provide helpful information for identifying previous SARS-CoV-2 infections, a lower cutoff than that of sample reactivity should be used. Anti-SARS-CoV-2 S/N IgM antibodies using conventional cutoffs seem useless for this purpose.

## Introduction

The assessment of anti-SARS-CoV-2 (severe acute respiratory syndrome coronavirus disease 2) antibodies is widely used as a surrogate measure of molecular test positivity for identifying previous SARS-CoV-2 infections across many different settings [1]. In particular, the quantification of anti-SARS-CoV-2 antibodies directed against the nucleocapsid protein (N) rather than vs. the spike (S) protein of the virus has been employed in many serological surveys in regions where coronavirus disease 2019 (COVID-19) vaccination is conducted with vaccines not containing the sequence (or part of) of the N protein, since the identification of anti-SARS-CoV-2 N antibodies would thus enable to identify the presence of an immune response against the virus and thus, theoretically, to classify the subjects as “infected” or “previously infected” by SARS-CoV-2 [2], [3], [4].

Nonetheless, several aspects (e.g., inconstant development and detection in subjects infected by new and highly mutated SARS-CoV-2 lineages, waning of serum levels over time, lack of validated “positivity” thresholds, etc.) contribute to raise reasonable doubts as to whether the assessment of anti-SARS-CoV-2 N antibodies may really considered a reliable marker of former natural infection in people who did not receive N-containing vaccines, especially when such results are used for guiding public health policies [5]. To this end, we planned this study to verify whether an immunoassay for quantifying anti-SARS-CoV-2 IgG/IgM antibodies against both spike (S) and nucleocapsid (N) proteins (i.e., S/N) may be used for identifying previous SARS-CoV-2 infections.

## Materials and methods

The study population consisted of a cohort of fully vaccinated healthcare workers from the Pederzoli Hospital (Peschiera del Garda, Verona, Italy), undergoing administration of Pfizer/Biontech mRNA BNT162b2 bivalent vaccine between November and December 2022. All study subjects underwent regular medical visits and molecular testing (Seegene Allplex SARS-CoV-2 Assay; Seegene Inc., South Korea or Altona Diagnostics RealStar SARS-CoV-2 RT-PCR Kit; Altona Diagnostics GmbH, Hamburg, Germany) for diagnosing SARS-CoV-2 infections every 2–4 weeks since 2020, as specifically described elsewhere [6]. Venous blood was drawn before bivalent COVID-19 vaccine administration, for measuring anti-SARS-CoV-2 antibodies with MAGLUMI 2019-nCoV lgG/IgM CLIA (SNIBE; Shenzhen, China). The test claims to detect the presence of either IgM or IgG antibodies concomitantly directed against both SARS-CoV-2 S and N proteins (i.e., S/N). Test results ≥1.1 (absorbance of sample/absorbance of calibrator) are considered reactive. According to a previous evaluation of these immunoassays, repeatability was found to be <6%, intermediate imprecision <6%, while the sensitivity for detecting acute SARS-CoV-2 infections was 1.00 for IgG and 0.88 for IgM, respectively [7]. Further analytical and technical details of these immunoassays are available elsewhere [7, 8].

All results were reported as median and interquartile range (IQR). The statistical analysis was conducted with Analyse-it (Analyse-it Software Ltd, Leeds, UK), with univariate (Spearman’s correlation, Mann–Whitney U test) and multivariate (multiple linear regression) analysis. All subjects recruited in this prospective observational study provided written informed consent. The study was conducted in accordance with the Declaration of Helsinki, and its protocol has been approved by the Ethics Committee of the Provinces of Verona and Rovigo (59COVIDCESC; November 8, 2021).

## Results

**Figure 1:**
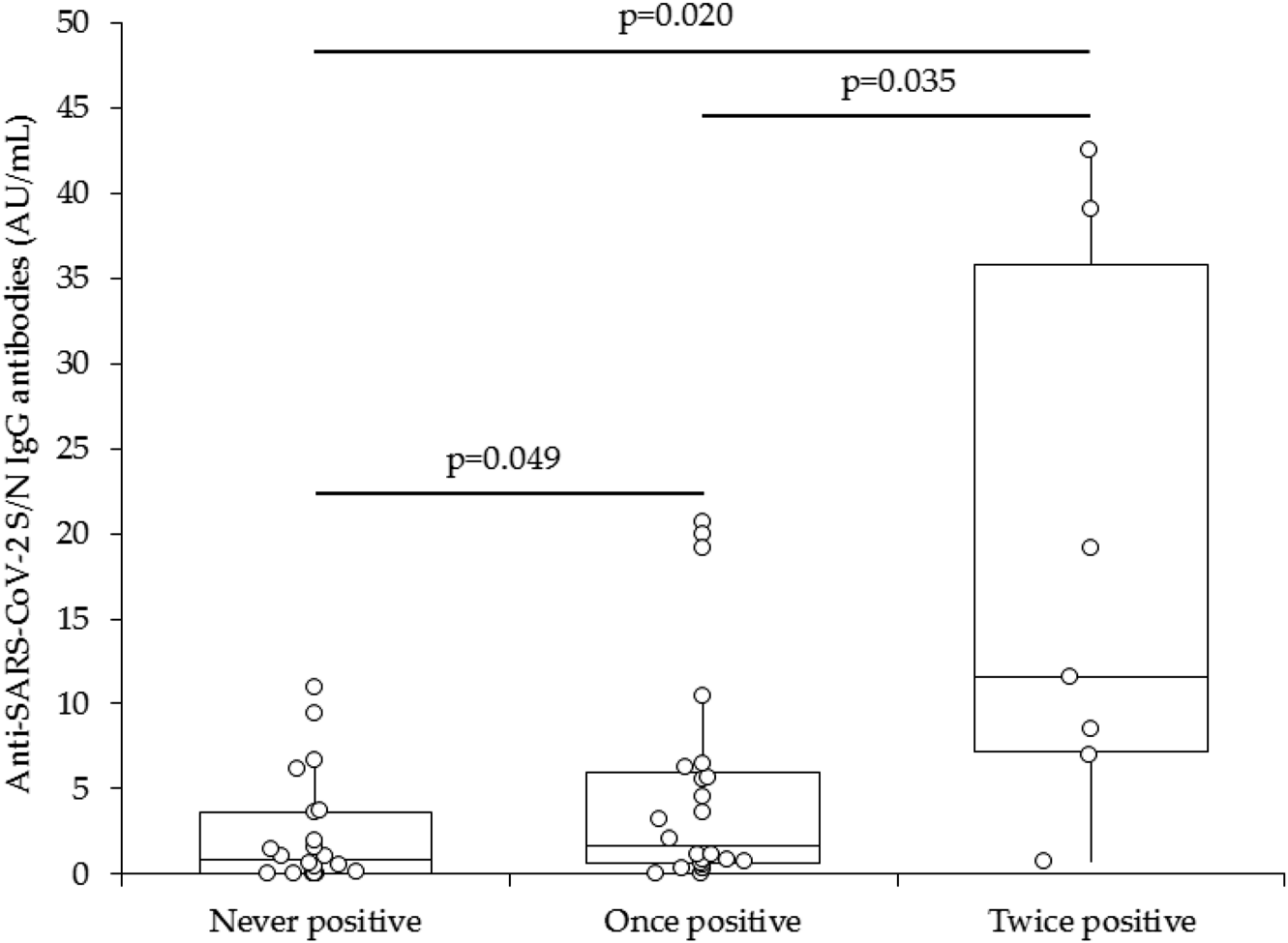
Anti-SARS-Cov-2 N IgG antibodies levels in subjects without positive molecular test result, one positive molecular test result, and two positive molecular test results for SARS-CoV-2 infection. SARS-CoV-2, severe acute respiratory syndrome coronavirus 2.

The final study population consisted of 53 subjects (median age, 43 years; IQR, 33–56 years; 27 females) (Table 1). Overall, 31 (58.5%) subjects had tested positive for SARS-CoV-2 by molecular testing between 2020 and 2022 (24 once, 7 twice). In univariate analysis, no correlation was found between anti-SARS-CoV-2 S/N IgM antibodies and molecular test result (r= −0.25; −0.48 to 0.03; p=0.074). Accordingly, the median values of anti-SARS-CoV-2 S/N IgM antibodies in subjects with (0.09; 95% CI, 0.01–0.15) or without (0.17; 95% CI, 0.09–0.23) molecular test positivity did not significantly differ (p=0.076). Molecular test positivity (r=0.33; 95% CI, 0.07–0.55; p=0.015) and the number of positive molecular test results (r=0.43; 95% CI, 0.18–0.63; p=0.001), but not the number of vaccine doses (r= −0.12; 95% CI, −0.38 to 0.16; p=0.392), were significantly correlated with anti-SARS-CoV-2 S/N IgG antibodies. Notably, neither age (0.21; 95% CI, −0.06 to 0.46; p=0.122), sex (r= −0.10; 95% CI, −0.36 to 0.18; p=0.476), nor body mass index (BMI; r= 0.19; IQR, −0.08 to 0.44; p=0.163) were associated with anti-SARS-CoV-2 S/N IgG antibodies. The associations between anti-SARS-CoV-2 S/N IgG antibodies and molecular test positivity or with the number of positive molecular tests remained statistically significant even in multiple linear regression analysis (p=0.029 and p<0.001, respectively) after adjusting for age, sex, body mass index (BMI), and vaccine doses. The median value of anti-SARS-CoV-2 S/N IgG antibodies gradually increased from subjects without molecular test positivity (0.84 AU/mL; IQR, 0.04–3.62 AU/mL), to those with one positive molecular test result (1.61 AU/mL; IQR, 0.61–5.99 AU/mL), and finally to those with two positive molecular test results (11.54 AU/mL; IQR, 7.20–35.77). The difference among all groups was always statistically significant (Figure 1).

**Table 1:** Main characteristics of the study population.

Parameter	Value
n	51
Age, years	43 (33–56)
Females	27 (50.9%)
BMI, kg/m^2^	24.7 (23.0–26.2)
Vaccine doses	
– One	4 (7.5%)
– Two	0 (0%)
– Three	49 (92.5%)
Previous SARS-CoV-2 RT-PCR positivity	31 (58.5%)
– Single positivity	24 (45.3%)
– Double positivity	7 (13.2%)
Anti-SARS-CoV-2 S/N IgM, AU/mL	0.11 (0.03–0.21)
Anti-SARS-CoV-2 S/N IgG, AU/mL	1.54 (0.46–6.42)

Values are reported as median and interquartile range (IQR) or percent. BMI, body mass index; SARS-CoV-2, severe acute respiratory syndrome coronavirus 2.

**Figure 2:**
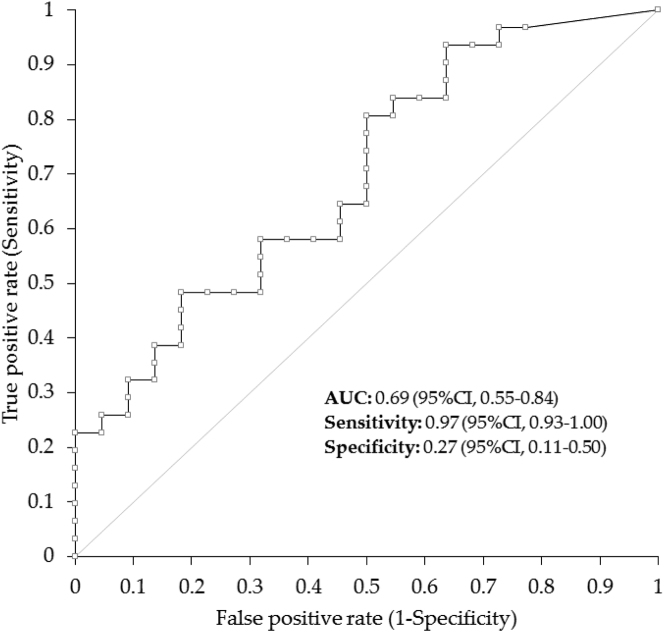
Receiving operating characteristics (ROC) curve analysis of anti-SARS-Cov-2 N IgG antibodies levels for identifying subjects with previous SARS-CoV-2 molecular test positivity. SARS-CoV-2, severe acute respiratory syndrome coronavirus 2.

In ROC curve analysis, anti-SARS-CoV-2 S/N IgG antibodies significantly predicted molecular test positivity (area under the curve [AUC], 0.69; 95% CI; 0.55–0.84; p=0.004), with the best cutoff of 0.05 AU/mL displaying 67.9% (95% CI, 53.7–80.1%) accuracy, 0.97 (95% CI, 0.93–1.00) sensitivity, and 0.27 (95% CI, 0.11–0.50) specificity (Figure 2). Notably, the accuracy, sensitivity, and specificity at the reactivity cutoff of anti-SARS-CoV-2 S/N IgG antibodies (i.e., ≥1.1 AU/mL) were 60.4% (95% CI, 46.0–73.6%), 0.65 (95% CI, 0.45–0.81), and 0.55 (95% CI, 0.32–0.76), respectively. A very high specificity vs. molecular test positivity could be reached by increasing the cutoff to 6.93 AU/mL, displaying 0.91 (95% CI, 0.17–0.51) specificity, 0.32 (95% CI, 0.17–0.51) sensitivity, and 56.6% (95% CI, 42.3–70.2%) overall accuracy (Table 2).

**Table 2:** Diagnostic accuracy, sensitivity, and specificity of anti-SARS-CoV-2 S/N IgG antibodies cutoffs for identifying subjects with molecular test positivity.

Cutoff	Accuracy	Sensitivity	Specificity
0.05 AU/mL	67.9% (95% CI, 53.7–80.1%)	0.97 (95% CI, 0.93–1.00)	0.27 (95% CI, 0.11–0.50)
1.10 AU/mL	60.4% (95% CI, 46.0–73.6%)	0.65 (95% CI, 0.45–0.81)	0.55 (95% CI, 0.32–0.76)
6.93 AU/mL	56.6% (95% CI, 42.3–70.2%)	0.32 (95% CI, 0.17–0.51)	0.91 (95% CI, 0.17–0.51)

SARS-CoV-2, severe acute respiratory syndrome coronavirus 2.

## Discussion

The generation of N (nucleocapsid)-specific antibodies, especially those belonging to the IgG class, is characteristic of patients in the convalescent phase of acute SARS-CoV-2 infection. The comprehensive analysis published by the Cochrane COVID-19 Diagnostic Test Accuracy Group has emphasized that anti-SARS-CoV-2 IgG antibodies develop in as many as 90% of all subjects recovering from an acute infection, while anti-SARS-CoV-2 IgM antibodies develop with a substantially lower frequency (i.e., around 70%) [4]. Thus, anti-SARS-CoV-2 IgG antibodies seem a globally more robust and reliable measure of acquired SARS-CoV-2 immunity than anti-SARS-CoV-2 N IgM antibodies. This is also reflected by the fact that anti-SARS-CoV-2 N IgM antibodies display a much faster decline over time compared to IgG, as clearly demonstrated by some previous studies [9, 10]. Thus, not only the development of anti-SARS-CoV-2 N IgM antibodies occurs less frequently than anti-SARS-CoV-2 N IgG antibodies, but also their values tend to decline faster over time, thus making them unreliable markers of previous SARS-CoV-2 infection. On the other hand, the anti-SARS-CoV-2 N IgG response seems more frequent and stable. Pushpakumara et al. reported that serum anti-SARS-CoV-2 N IgG antibodies are detectable in the vast majority of subjects with previous natural infection or vaccination with whole virus inactivated vaccines [11]. In a recent study, Dhakal et al. studied 182 adult participants (101 vaccinated and then infected, 28 infected and then vaccinated, and 53 never vaccinated, respectively) [12], and reported that patients infected by SARS-CoV-2 before vaccination or never vaccinated had persistently increased anti-SARS-Cov-2 N IgG antibodies levels, while those vaccinated before being infected displayed significantly lower values of these antibodies (p<0.001 in each case). Nonetheless, another prospective study found that the level of anti-SARS-CoV-2 N IgG antibodies seem to decrease earlier than those of anti-SARS-CoV-2 S IgG antibodies, especially in older people [13], thus raising important doubts as to whether this test may have enough sensitivity to identify subjects with previous SARS-CoV-2 infection.

Taken together, the results of the present study provide important answers to these questions. First, we did not find a significant association between anti-SARS-CoV-2 S/N IgM antibodies and molecular test positivity (their median values were also found to be paradoxically lower in subjects with molecular test positivity than in those without), thus allowing us to rapidly exclude this test from further analyses due to its highly unreliable clinical significance. Then, we showed that the reactivity cutoff value of anti-SARS-CoV-2 S/N IgG antibodies (i.e., 1.1 AU/mL), which has been specifically designed for detecting the humoral immune response during or immediately after an acute infection, failed to display adequate diagnostic performance for the purpose of detecting molecular test positivity over a nearly 3-year period. Specifically, the overall accuracy and sensitivity for identifying subjects with previous infection (i.e., molecular test positivity) were 60.4% and 0.65, while a much lower cutoff (i.e., 0.05 AU/mL) enabled to increase accuracy and sensitivity to 67.9% and 0.97, respectively. Yet, this increased sensitivity was achieved at the expense of the specificity, which dropped from 0.55 to 0.27. This actually means that the lower 0.05 AU/mL cutoff would enable to identify virtually all subjects with a previous SARS-CoV-2 infection, but would be plagued by a considerable rate of false positive cases (i.e., around 35%). On the other hand, the use of a higher cutoff (i.e., 6.93 AU/mL) would enable to rule out previous infections (i.e., molecular test positivity) with very high specificity (i.e., over 0.90), though this would be plagued by a considerable rate of false-negative cases (i.e., 51%). In keeping with these findings, Schaffner et al. studied the trajectory of anti-SARS-CoV-2 N IgG antibodies over time in 82 subjects with a laboratory-defined SARS-CoV-2 infection and found that the sensitivity using the cutoff index suggested by the manufacturer declined from 0.91 to 0.63 at 48 and 140 days postinfection, respectively [14]. This is consistent with the evidence emerged from multiple other clinical studies, such as that published by Krutikov et al. [15], who identified a median time to reversion of anti-SARS-CoV-2 N IgG antibodies of around 242 days, thus meaning that over half of subjects would no longer display “reactive” values of these antibodies 1 year after an acute infection. Similarly, substantial seroreversion of anti-SARS-CoV-2 N total antibodies was observed at 18 months postinfection in a longitudinal study conducted by Loesche et al. [16].

More than 3 years after the pandemic has commenced, the diagnostic approach to COVID-19 remains challenging. Although it has been universally agreed that anti-SARS-CoV-2 antibodies provide only modestly useful clinical information for diagnosing an acute infection, their role in monitoring the humoral response remains highly valuable [17]. Nonetheless, when used in serological surveys, we suggest that “mobile” cutoffs of anti-SARS-CoV-2 N IgG antibodies shall be identified depending on the method and purpose of the test, with lower thresholds adopted for identifying subjects with previous SARS-CoV-2 infection, and higher thresholds adopted for identifying those without previous SARS-CoV-2 infection, respectively.

In conclusion, although anti-SARS-CoV-2 S/N IgG antibodies provides helpful information for identifying previous SARS-CoV-2 infections, a lower cutoff than that of sample reactivity should be used, as earlier suggested by others [18]. Anti-SARS-CoV-2 S/N IgM antibodies appear almost useless for this purpose.
